# Effects of Transition Metal Substituents on Interfacial and Electronic Structure of CH_3_NH_3_PbI_3_/TiO_2_ Interface: A First-Principles Comparative Study

**DOI:** 10.3390/nano9070966

**Published:** 2019-07-01

**Authors:** Yao Guo, Yuanbin Xue, Xianchang Li, Chengbo Li, Haixiang Song, Yongsheng Niu, Hu Liu, Xianmin Mai, Jiaoxia Zhang, Zhanhu Guo

**Affiliations:** 1 Department of Chemical and Environmental Engineering, Anyang Institute of Technology, Anyang 455000, China; 2 Department of Mathematics and Physics, Anyang Institute of Technology, Anyang 455000, China; 3 Integrated Composites Laboratory (ICL), Department of Chemical & Biomolecular Engineering, University of Tennessee, Knoxville, TN 37996, USA; 4 Key Laboratory of Materials Processing and Mold (Zhengzhou University), Ministry of Education; National Engineering Research Center for Advanced Polymer Processing Technology, Zhengzhou University, Zhengzhou 450002, China; 5 School of Urban Planning and Architecture, Southwest Minzu University, Chengdu 610041, China; 6 School of Material Science and Engineering, Jiangsu University of Science and Technology, Zhenjiang 212003, China

**Keywords:** organic-inorganic perovskites, interface, first-principles calculations

## Abstract

To evaluate the influence of transition metal substituents on the characteristics of CH_3_NH_3_PbI_3_/TiO_2_, we investigated the geometrical and electronic properties of transition metal-substituted CH_3_NH_3_PbI_3_/TiO_2_ by first-principles calculations. The results suggested that the substitution of Ti^4+^ at the five-fold coordinated (Ti_5c_) sites by transition metals is energetically favored. The substituted interface has enhanced visible light sensitivity and photoelectrocatalytic activity by reducing the transition energies. The transition metal substitution can effectively tune the band gap of the interface, which significantly improves the photo-reactivity. The substituted systems are expected to be more efficient in separating the photo-generated electrons-holes and active in the visible spectrum.

## 1. Introduction

Hybrid halide perovskites as light harvesters have been the focus of the photovoltaic field over the past years owing to their impressive power conversion efficiency (PCE) and promising commercial applications [1,2]. The CH_3_NH_3_PbI_3_ perovskites dominate this field and have been studied extensively [3]. The typical device architecture of perovskite solar cells (PSC) is composed of the TiO_2_-based electron transport layer (ETL), the perovskite (CH_3_NH_3_PbI_3_) absorber-based layer, the spiro-OMeTAD hole transport layer (HTL) and the corresponding electrodes [4]. The perovskite/ETL interface plays an important role in determining the charge separation and transport properties as well as the PSC device performance, which has been widely explored for many years [5,6,7,8].

TiO_2_ is a good candidate material due to its chemical stability, high charge transport property and low cost [9]. The electron can be effectively transported from CH_3_NH_3_PbI_3_ to the TiO_2_ layer because the conduction band of TiO_2_ is lower than that of CH_3_NH_3_PbI_3_ [10]. Generally, the ultra-thin compact TiO_2_ layer is prepared at a high temperature (over 450 °C) [11]. It seriously undermines the electrical properties of the ETL in conductivity, mobility, and electronic trap states, thereby affecting the efficiency and stability of PSC. Elemental substitution in the compact TiO_2_ layer is an effective solution to improve the electrical properties and device performance [12,13,14,15,16,17,18,19,20,21]. Transition metal substitution [22] is an effective approach to enhance the photocatalytic activity of TiO_2_ due to their unique *d* electronic configuration and spectral characteristics. According to previous studies [13,14,15,16,17,18,19,20,21], a wide range of substitutional elements such as Zn^2+^, Y^3+^, Nb^5+^, Ru^4+^ and W^6+^ have been investigated in TiO_2_. Research has made remarkable progress in identifying that the substitution of the TiO_2_ layer by the transition metal in PSC is an effective mean to improve the photocurrent and electron-hole recombination [14,15,16,17,18]. Substituents in TiO_2_ film can improve the electrical characteristics of ETL, which promote PCE and stability of PSC [13,23]. Therefore, transition metal substituents in the TiO_2_ layer are quite important and further characterizations are required to understand the effects of substituents in the CH_3_NH_3_PbI_3_/TiO_2_ interface. Although the electronic properties of perovskite/ETL interface have been widely investigated by experiments and density-functional-theory (DFT) calculations [24,25,26,27,28,29,30,31], the existence of theoretical studies aiming to understand the fundamental role of the interfacial substituents is still rather scarce. In addition to the primary experiments, the first-principles DFT calculations are highly important to acquire further knowledge concerning the effects of transition metal substitution and contribute to new strategies for interface optimization. The main contributions of our study are helpful to draw guidelines for substitution mechanism of the CH_3_NH_3_PbI_3_/TiO_2_ interface, thus enhancing the photovoltaic performance in PSC.

## 2. Methods

The Vienna *ab initio* simulation package (VASP) [32] was employed as the first-principles calculations platform. The computer software program is based on the DFT approach using plane wave basis within a periodic boundary condition. The projected augmented wave (PAW) [33] pseudopotentials were applied for efficient computation. The exchange and correlations items were treated within the framework of generalized-gradient approximation (GGA) of Perdew-Burke-Ernzerhof (PBE) [34]. A plane wave basis cutoff energy of 500 eV was used. Integrations in reciprocal space were sampled using the Monkhorst-Pack grids [35] with a minimum spacing of 0.2 Å^−1^. Convergence criteria were set as 1.0^−6^ eV in total energy and 1.0^−2^ eV/Å in atomic force, respectively. Recent theoretical studies indicated that the *GW* (Green’s function *G* with screened interaction *W*) and hybrid functional approach can provide an accurate description of the electronic structures [36,37]. Fortunately, DFT was able to qualitatively reproduce the *GW* trend. Hence, we performed GGA+*U* calculations on the CH_3_NH_3_PbI_3_/TiO_2_ interfaces with reasonable computational cost. Based on previous research and experiences [38,39,40,41,42,43], the GGA+*U* approach with the on-site Coulomb interaction correction predicted band gap correctly. The values of parameter *U* were 6 eV for the Ti^4+^ 3*d* orbit and 4 eV for the *d*-orbits of transition metal substituents. Gaussian broadening [44] with half-width of 0.1 eV for the electronic eigenvalues was used to accelerate the convergence in the *k*-point sum. The dipole correction was included because the interface configuration does not have mirror symmetry along the *c*-axis. The spin orbit coupling (SOC) effect [45] was not included because it was negligible in the geometry. The atomic structures shown were produced by using the visualization for electronic and structural analysis (VESTA) program [46].

**Figure 1:**
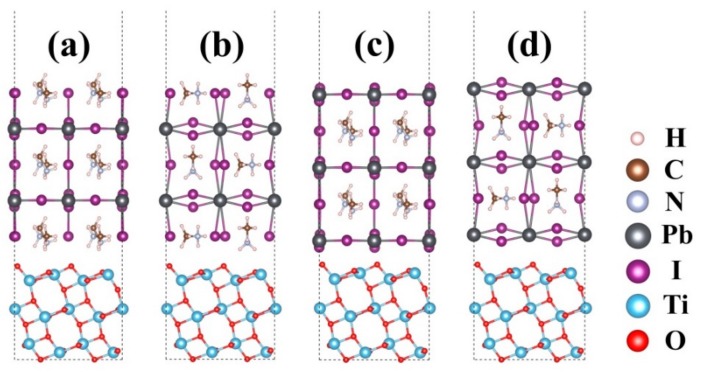
Schematic illustration of pristine CH_3_NH_3_PbI_3_/TiO_2_ interface models: (**a**) CH_3_NH_3_I/TiO_2_ (**b**) CH_3_NH_3_I/TiO_2_ with rotation (**c**) PbI_2_/TiO_2_ (**d**) PbI_2_/TiO_2_ with rotation.

**Figure 2:**
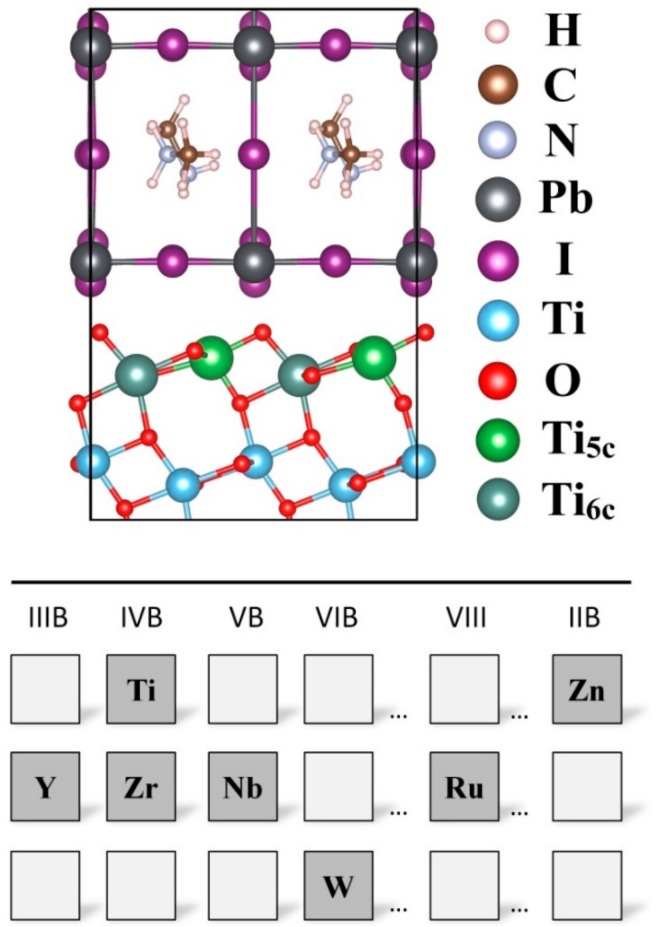
Schematic illustration of the transition metal substituents at Ti_5C_ and Ti_6C_ sites of the CH_3_NH_3_PbI_3_/TiO_2_ interface.

According to the experimental results [47], there is an ordered lattice structure existing on the CH_3_NH_3_PbI_3_/TiO_2_ interface. The (110) slab of the CH_3_NH_3_PbI_3_ nanocrystal coordinated with the (101) slab of anatase TiO_2_, forming an ordered lattice structure at the interface. The lattice mismatch between CH_3_NH_3_PbI_3_ (110) and TiO_2_ (101) has been evaluated in previous studies [48]. It was found that using the experimental results of the CH_3_NH_3_PbI_3_ (110) surface, the band-gap only varies slightly, with a corresponding total energy decrease, suggesting that a minimal strain is introduced by the lattice mismatch between the two materials. In spite of a relatively large lattice mismatch, we selected the experimental interface [47] between CH_3_NH_3_PbI_3_ (110) and TiO_2_ (101) to carry out the investigation. To construct the aspired interface structures, the CH_3_NH_3_PbI_3_ (110)/TiO_2_ (101) interface was chosen as our objective due to the experimental results [47] and stability of the corresponding surface. The (2 × 2) supercell of the CH_3_NH_3_PbI_3_ (110) and the (1 × 3) supercell of the anatase TiO_2_ (101) were employed to decrease the misfit. The interface model employed the average size of both CH_3_NH_3_PbI_3_ and TiO_2_ slabs to make a small mismatch. The CH_3_NH_3_PbI_3_ perovskite was composed of the CH_3_NH_3_I and PbI_2_ units along the [001] direction; therefore, both CH_3_NH_3_I and PbI_2_ terminations using five-layer slabs were considered in this work. The anatase (101) supercell contains 36 TiO_2_ units or 108 substrate atoms. The interfaces were built by connecting the CH_3_NH_3_PbI_3_ (110) slab with the anatase (101) slab and leaving a 20 Å vacuum gap in the perpendicular direction. A schematic representation of the interfacial system used in this work is presented in Figure 1. In fact, the TiO_2_ was the substrate to grow perovskite films; hence, apart from the bottom two Ti and four O layers, which were maintained in their ideal bulk positions, all atomic coordinates of the others layers were fully relaxed. The transition metal-substituted anatase (101) surface model was constructed by substituting the surface Ti^4+^ with substituent atoms. As shown in Figure 2, there are two possible surface sites (five/six-fold coordinated Ti^4+^ cation, hereafter abbreviated as Ti_5C_ and Ti_6C_) for the substituent atoms to replace [49]. Till date, the atomic arrangement of the CH_3_NH_3_PbI_3_/TiO_2_ is still unclear due to limit of the experimental techniques. Based on previous studies of the transition metal-substituted TiO_2_ surface [50,51], the interfacial configurations were carefully designed to make the substituent effect more prominent at the interface region. We assume that all six different transition metal ions partially substituted at the Ti^4+^ sites (Ti_5C_ or Ti_6C_) correspond to the substitution concentration of 17% and the supercell is represented by Ti_0.83_M_0.17_O_2_ (*M* = Zn^2+^, Y^3+^, Zr^4+^, Nb^5+^, Ru^4+^, W^6+^). The substituents can be classified as 3*d* transition metal (Zn^2+^), 4*d* transition metal (Y^3+^, Zr^4+^, Nb^4+^, Ru^4+^) and 5*d* transition metal (W^6+^) ions.

## 3. Results and Discussions

As shown in Table 1, the optimized 0 K DFT lattice parameters of anatase TiO_2_ crystal are *a* = 3.79 Å and *c* = 9.53 Å, which is in agreement with previous experiments [52]. The atomic positions of tetragonal CH_3_NH_3_PbI_3_ are based on the results of the previous report [53,54]. The 0 K DFT lattice parameters are *a* = 8.80 Å and *c* = 13.05 Å. The strong interfacial interaction in CH_3_NH_3_PbI_3_/TiO_2_ is mainly through iodine and under-coordinated titanium atoms. The stability of the selected interface can be evaluated by comparing the calculated binding energies [55,56]. The calculated binding energies of different interfaces are listed in Table 2, together with lattice mismatch. The lattice mismatch between CH_3_NH_3_PbI_3_ and TiO_2_ was −12.0% and −13.8%, respectively. The interfacial binding energies and lattice mismatch can be predicted by the following equations [25]:

*E*_binding_ = *E*_anatase_ + *E*_perovskite_ − *E*_total_ (1)

*M_perovskite/anatase_* = (*a_perovskite_* − *a_anatase_*)/*a_perovskite_* (2)

where *E*_total_, *E*_anatase_, and *E*_perovskite_ are the corresponding energies of the interface, anatase and perovskite surfaces, respectively. The *a_anatase_* and *a_perovskite_* represent the lattice parameter of TiO_2_ and CH_3_NH_3_PbI_3_, respectively. Zero energy corresponds to the energetically less-stable structure. It is not surprising that the perovskite/TiO_2_ interfaces without rotation are more stable than their corresponding rotated ones. This could be because of the difference in lattice mismatches. The strain may affect the interfacial stability between perovskite and TiO_2_. For rotation-free interfaces, the *E*_binding_ of the two systems is quite similar. The CH_3_NH_3_^+^ cation interacted with TiO_2_ partially containing weak van der Waals (vdW) interactions. In contrast, the interaction between Pb^2+^ cation and TiO_2_ leads to the formation of stable chemical bonds. This character is similar to previous works [25,54]. The interfacial structures of the relatively stable rotation-free perovskite/TiO_2_ are adopted in subsequent calculations. The interface supercell lattice parameters are given by *a* = 10.95 Å, *b* = 11.64 Å and *c* = 49.95 Å.

To explore the influence of transition metal substitution on the interfacial stability of the CH_3_NH_3_PbI_3_/TiO_2_ interface structure, the Nb-substituted interfaces were systematically investigated. The dependence of Nb^5+^ substituent on the depth within the interface layers were evaluated based on the total energies (seen Table 3). The six-coordinated Ti_6C_ substituted interface with the lowest total energy is accepted as the most stable configuration. Despite the cleaved and unsaturated bond, the five-coordinated Ti_5C_ substituted interfaces still show relative low total energy. The Nb^5+^ substituent was energetically favorable at the Ti_5C_ and Ti_6C_ sites of the TiO_2_ surface. Therefore, to characterize the effect of substitution at the CH_3_NH_3_PbI_3_/TiO_2_ interface, both Ti_5C_ and Ti_6C_ substitution sites are considered in subsequent calculations. The substitution of Nb^5+^ at both Ti_5C_ site and Ti_6C_ sites in the top TiO_2_ layer was considered. For each situation, two different configurations, namely CH_3_NH_3_I/TiO_2_ and PbI_2_/TiO_2_, were considered in this study. The calculated interfacial binding energies and Bader charge were listed in Table 4. The interfacial binding energies become stronger after substitution, which indicates that substituting Ti^4+^ with Nb^5+^ could enhance the stability and strength of the perovskite/TiO_2_ interface. The CH_3_NH_3_I/TiO_2_ interface has a larger energy than the PbI_2_/TiO_2_ interface. The degree of charge transferring is evaluated by the Bader charge analysis [57]. The negative value means the transfer of excess electrons from the perovskite to TiO_2_ because the perovskite layer has a higher average potential than the TiO_2_ layer. As can be seen in Table 4, there is less charge transfer in the PbI_2_/TiO_2_ than that of the CH_3_NH_3_I/TiO_2_. This can be explained by the fact that the PbI_2_ layers have a relatively lower potential than the CH_3_NH_3_I layers. Moreover, it also can be seen that the Nb^5+^ substituents located at the Ti_5C_ site have a larger charge transfer than that of the Ti_6C_ site. Liu et al. reported that the potential drop on the CH_3_NH_3_I/TiO_2_ is deeper than that of the PbI_2_/TiO_2_ [25]. As a result, a strong accumulation region can be formed at the CH_3_NH_3_PbI_3_/TiO_2_ interface, leading to a better electron-hole separation in the PSC. To emphasize and compare the influence of more different transition metal substituents on the CH_3_NH_3_PbI_3_/TiO_2_ interface, we make the approximation that only the Ti_5C_-substitute interface will be considered in subsequent investigations.

**Figure 3:**
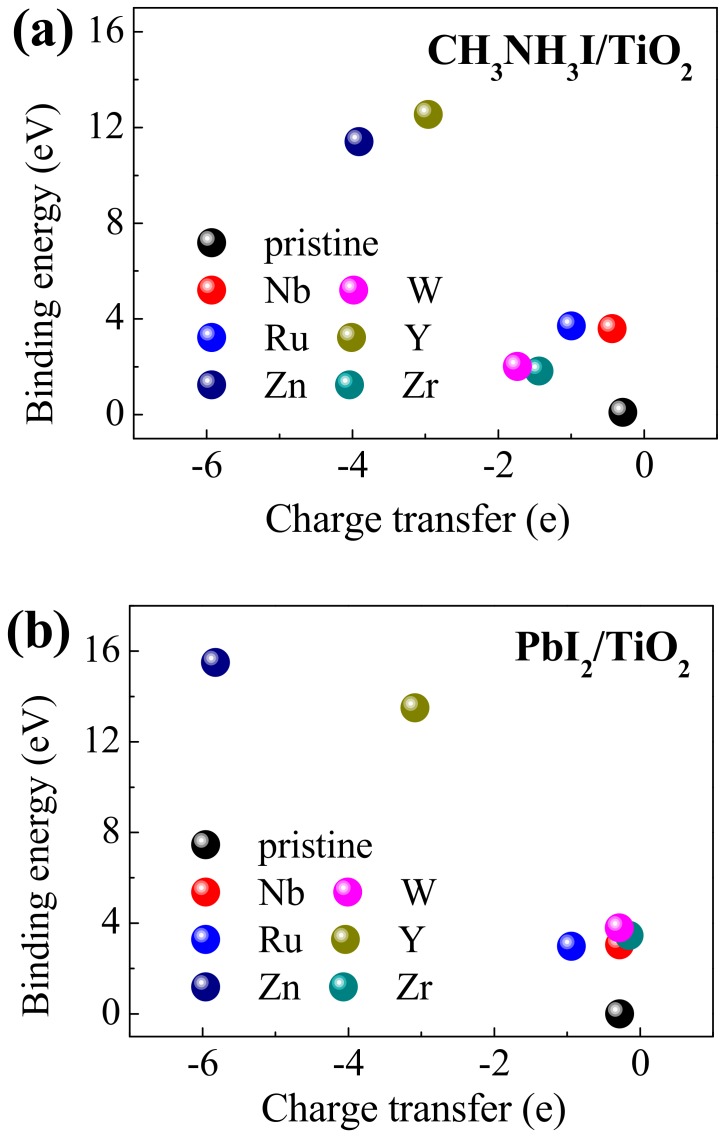
Relationship between the interfacial charge transfers and binding energies of transition metal-substituted CH_3_NH_3_PbI_3_/TiO_2_: (**a**) CH_3_NH_3_I/TiO_2_ (**b**) PbI_2_/TiO_2_.

Both the interfacial charge transfers and *E*_binding_ of the transition metal-substituted CH_3_NH_3_PbI_3_/TiO_2_ interfaces in Figure 3 were combined to evaluate the influence of transition metal substitution in the PSC. Transition metals can be divided into three types: n (Nb^5+^, W^6+^), p (Zn^2+^, Y^3+^) and isovalent (Zr^4+^, Ru^4+^) substitutions. Zero energy (pristine) corresponds to the energetically less-stable structure. It can be seen that the transition metals have different *E*_binding_ while the values of each substituted interface are positive. It also can be discerned clearly from Figure 3 that the transition metal-substituted interface has a much higher binding energy. This suggests that substituting transition metals *M* (*M* = Zn^2+^, Y^3+^, Zr^4+^, Nb^5+^, Ru^4+^, W^6+^) for Ti^4+^ at the interface layer could significantly enhance the interface strength between perovskite and TiO_2_. In addition, Figure 3 displays the comparison of charge transfers at the perovskite/TiO_2_ interface substituted with various transition metals. One can clearly see that the charge transfer in the interface becomes larger with the addition of transition metals. It should be pointed out that Zn^2+^ and Y^3+^ substitution for Ti^4+^ at the interface layer is energetically favorable in terms of binding energy (16.6 and 13.5 eV) and charge transfer (−5.8 and −3.1 e) for the PbI_2_/TiO_2_ interface. This enhancement can be attributed to the optimized energy band alignment, which could improve the electron transfer behavior between ETL and perovskite. The theoretical results can provide support for future experimental design and synthesis of a stable perovskite/TiO_2_ interface, possessing strong electron transfer capacity. Due to their relatively stronger binding energies, the interfacial structure of PbI_2_/TiO_2_ is selected for subsequent investigations.

**Figure 4:**
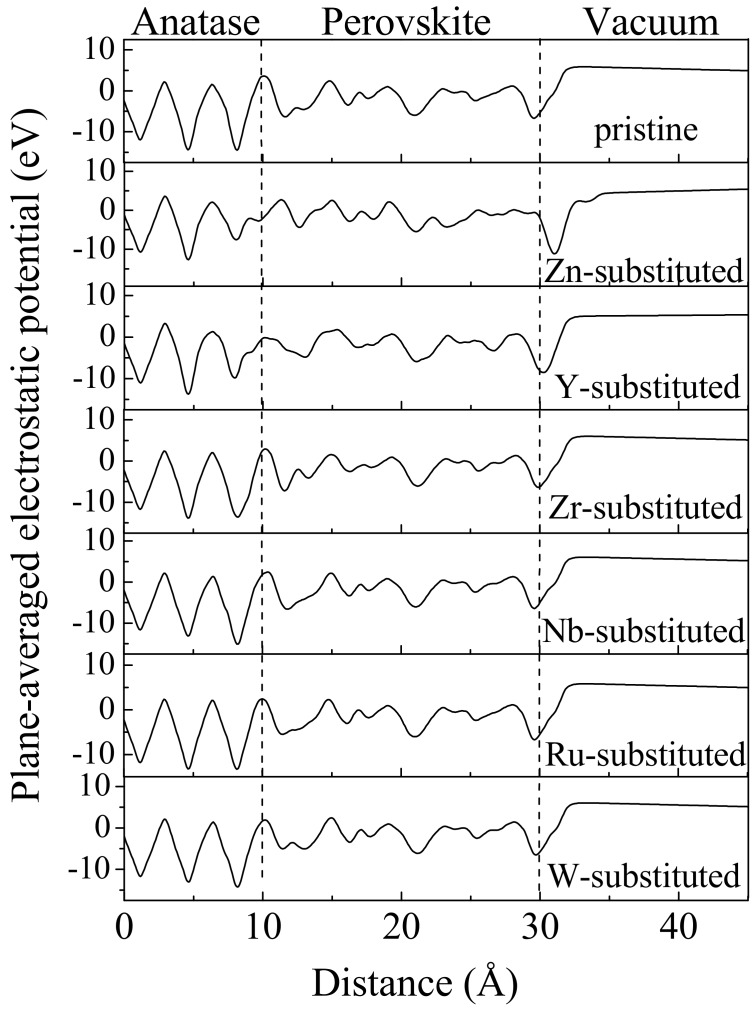
Planar averaged electrostatic potential across the optimized transition metal-substituted CH_3_NH_3_PbI_3_/TiO_2_.

The degree of the total potential drop across the CH_3_NH_3_PbI_3_/TiO_2_ interfaces reliably indicates their photo-excited charge separation capabilities [58]. To clearly show their difference, the planar averaged electrostatic potential of the seven perovskite/TiO_2_ heterostructures was calculated to estimate the electronic level positions (Figure 4). The Fermi level differences between CH_3_NH_3_PbI_3_ and TiO_2_ build the driving force for the electron to transfer from the CH_3_NH_3_PbI_3_ to the TiO_2_ slab. Actually, a substantial amount of charge gather at the TiO_2_ side due to the abrupt potential drop near the interface. Then, the built-in electric field in the interface hampers more electron transfer across the interface, and the electronic charge transfer equilibrium is reached. It is known that the built-in electric fields originate from the surface-surface interactions, particularly for the Pb^2+^ movement and the CH_3_NH_3_^+^ orientation [58]. The incorporation of transition metals *M* (*M* = Zn^2+^, Y^3+^, Zr^4+^, Nb^5+^, Ru^4+^, W^6+^) enhances the polarization and the built-in electric field across the interfacial heterostructure. As shown in Figure 4, it is worth mentioning that the Zn- and Y-substituted interface models (*x* = 10Å) exhibit a substantial slope of electrostatic potential, while the others show the parameters’ electrostatic potential. The potential drop in the Zn- and Y-substituted interfaces is notably steeper than that in the other interfaces, which serves as a reservoir for electrons. Hence, a substantial number of electrons gather at the TiO_2_ surface, implying that the Zn^2+^ and Y^3+^ substituents are more efficient in separating the photo-generated electrons and holes.

**Figure 5:**
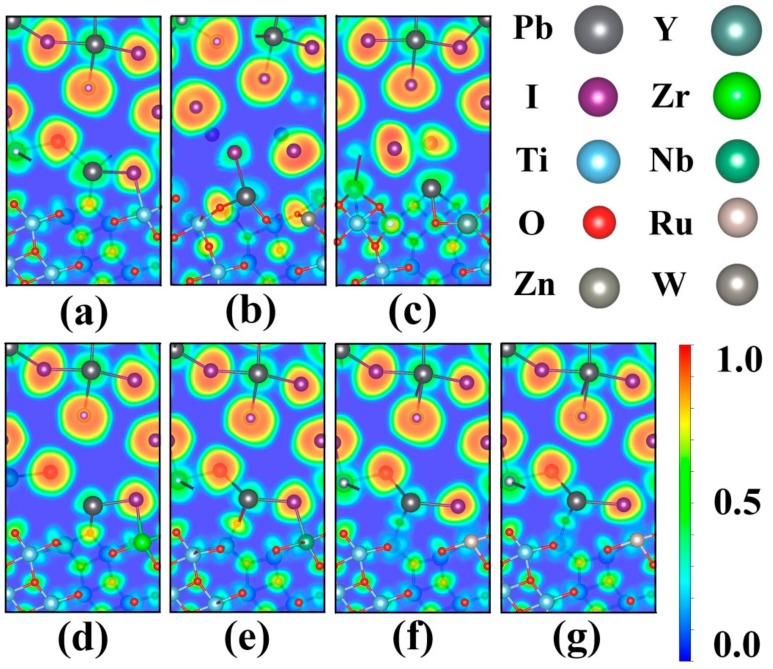
ELF of the optimized CH_3_NH_3_PbI_3_/TiO_2_ interfaces at (010) plane: (**a**) pristine, (**b**) Zn-substituted, (**c**) Y-substituted, (**d**) Zr-substituted, (**e**) Nb-substituted, (**f**) Ru-substituted, (**g**) W-substituted.

**Figure 6:**
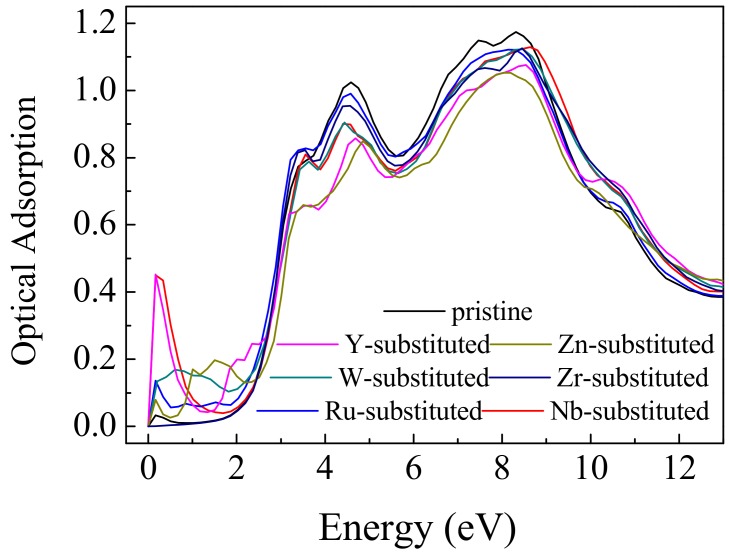
Comparison of the optical absorption of the transition metal-substituted CH_3_NH_3_PbI_3_/TiO_2_ interfaces.

To scrutinize the electronic property differences in various transition metal-substituted CH_3_NH_3_PbI_3_/TiO_2_, the bonding characteristics were analyzed by electron localization function (ELF), which can illustrate the type of bonding and delocalization of electron density in the interfacial system [59]. Figure 5 depicts the interfacial structures and ELF contour plots at (010) planes crossing the Pb^2+^ and I^−^ with color scheme for various interfacial systems. The ELF ranges from 0 to 1, where red corresponds to a full localization, blue indicates a full delocalization, and green implies the uniform electron gas. The ELF slice exhibited lesser electron localization for the transition metal substituents than Ti^4+^, which indicates a more covalent nature of the substituent-O interaction compared to the Ti–O interactions [60,61]. As can be seen, Figure 6a,d–g are quite similar, which explains why there is no variation in their geometry. By contrast, substitution with Zn^2+^ and Y^3+^ produced larger geometrical modification and electronic change in the CH_3_NH_3_PbI_3_/TiO_2_ interface. The ionic radius of transition metal substituents explains why there is no geometrical change with regard to the pristine and substituted systems. Compared with the Ti^4+^ cation (~0.6 Å) [62], the relatively large ionic radius of Y^3+^ (~0.9 Å) [63] and Zn^2+^ (~0.7 Å) [63] leads to distortions in the CH_3_NH_3_PbI_3_/TiO_2_ interface. Moreover, the binding energy and charge transfer analysis also led to the same conclusion. Interfacial bond strength varied with the ionic radius of transition metal in the substituted interfacial systems.

The optical properties, including optical reflectivity, refractive index and absorption efficient can be obtained by dielectric function [64]. Taking into account that the PCE of CH_3_NH_3_PbI_3_ mostly originates from the efficient use of visible light in the solar spectrum, only the calculated electronic absorption spectra of the CH_3_NH_3_PbI_3_/TiO_2_ interfaces have been investigated. The optical absorption coefficients (α) of different interfaces based on the obtained electronic structures are presented and compared in Figure 6. The absorption spectra parallel to *x*-axis were selected to examine the influence of transition metal substituents on the optical properties. The shapes of each absorption curve are close. As depicted in Figure 6, the CH_3_NH_3_PbI_3_/TiO_2_ interfaces have two absorption peaks—around 3.5 eV and 7.5 eV. The peak around 3.5 eV mainly comes from the conduction-to-valence band transition from I 5*p* or Pb 6*s* states to Pb 6*p* states [65]. On the other hand, the peak around 7.5 eV can be ascribed to the intrinsic band gap of pristine TiO_2_ and the electron shifting from the O 2*p* to Ti 3*d* orbitals [66]. It has also been reported that the absorption of pure TiO_2_ is limited to ultraviolet (UV) light and exhibits inefficient response for visible light. In case of the pristine CH_3_NH_3_PbI_3_/TiO_2_, our theoretical calculation is consistent with the experimental values and theoretical studies [67,68]. In case of the substituted CH_3_NH_3_PbI_3_/TiO_2_, the substituted interface still shows poor photoactivity in the visible-light region for solar light harvesting. However, it has an extra absorption peak in the low energy region (less than 2 eV). The incorporation of the transition metal substituents into the CH_3_NH_3_PbI_3_/TiO_2_ interface leads to an obvious red-shift effect. The distinct absorption peak at 0.3 eV in the low energy region can be attributed to the band gap near the Fermi level. The decrease in intensity of transition energies is caused by the split intra-band transitions between the impurity states, rendering the more obvious red-shift [68]. Compared with the pristine CH_3_NH_3_PbI_3_/TiO_2_ interface, the transition metal-substituted CH_3_NH_3_PbI_3_/TiO_2_ interface is expected to be more active for efficient visible-light photo-catalysis.

**Figure 7:**
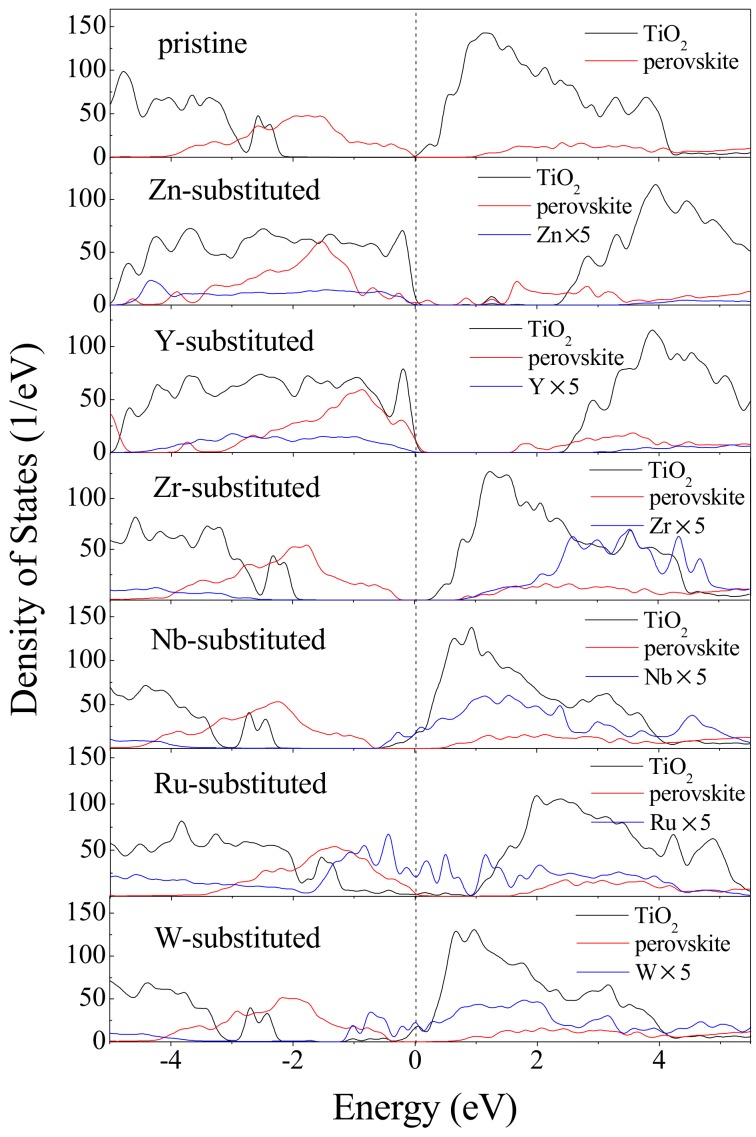
DOS of pristine and transition metal-substituted CH_3_NH_3_PbI_3_/TiO_2_ interface.

In order to further elucidate the charge carrier separation mechanism of the CH_3_NH_3_PbI_3_/TiO_2_ interfaces, partial density of states (PDOS) have been calculated for pure and substituted CH_3_NH_3_PbI_3_/TiO_2_ interfaces. As seen in Figure 7, the PDOS is split into contributions from CH_3_NH_3_PbI_3_, TiO_2_ and transition metal substituents. The substituent component is magnified five times for better visibility. It is already known that the band gap of TiO_2_ is wider than that of the CH_3_NH_3_PbI_3_ perovskite [25,26]. Besides, the conduction band minimum (CBM) of TiO_2_ is lower than that of CH_3_NH_3_PbI_3_. They can excite electrons from the valence band of CH_3_NH_3_PbI_3_ (I 5*p* and Pb 6*s* orbitals) to conduction band of CH_3_NH_3_PbI_3_ (Pb 6*p*), and then transfer to conduction band of TiO_2_ (Ti 3*d*). The difference between Pb 6*p* and Ti 3*d* decided the efficiency of charge transfer across the interface [69]. The interfacial band gaps can be tuned by n, p, and isovalent substituents using the selected transition metals. As shown in Figure 7, n substitution agents such as Nb^5+^ and W^6+^ pushed the Fermi level into the conduction band and made the system metallic. The intensity of the Fermi levels entering into the conduction band should be increased as the *d* states of substituents changes from 4*d* to 5*d* transition metals. By contrast, in the p substitution agents (Zn^2+^ and Y^3+^) modified interfaces, the Fermi levels shifted from valence band to conduction band, leading to obvious band gaps compared with the pristine system. For the isovalent substituted (Zr^4+^, Ru^4+^) interfaces, the PDOS shape of substituted interface is wider than that of the pristine interface, which implies that the electronic nonlocality becomes quite obvious. The delocalized transition metal *d* state contributes to the electron–hole pair separation in the PSC and supports carrier migration within the photo-catalysis process.

**Figure 8:**
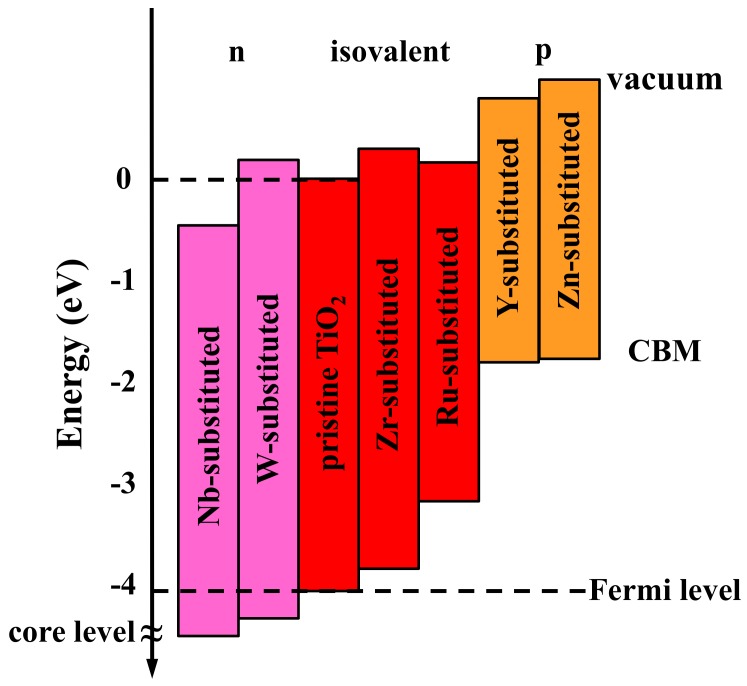
Schematic energy level diagram of element substituted TiO_2_.

Schematic diagrams of the energy levels of various elements’ substitution TiO_2_ are shown in Figure 8. The vacuum level of the system was set to 0 eV for comparison. The CBM of pristine TiO_2_ was found to be located at −4.1 eV. It can be seen that the substituent ion effectively modified the CBM state of the TiO_2_. In all the cases, the CBM gradually up-shifts to the vacuum level as the substituent change from n to p. The Fermi level shifts downward with p substituent and the electron injection from perovskite to p substituted TiO_2_ will be hindered. On the other hand, the optimal band alignment between perovskite and n substituted TiO_2_ could effectively improve the charge transport and suppress charge recombination. From this point of view, Nb^5+^ and W^6+^ substituted interfaces are expected to have better device performance. Tuning energy level alignment by element substitution (*M* = Zn^2+^, Y^3+^, Zr^4+^, Nb^5+^, Ru^4+^, W^6+^) is confirmed to be an effective way to optimize charge transportation and thus enhance the PCE of PSC.

## 4. Concluding Remarks

First-principles computations were utilized to characterize the structural, electronic and optical properties of the transition metal-substituted CH_3_NH_3_PbI_3_/TiO_2_ interface. Through density functional calculations for binding energy and charge transfer of various configurations, we found that the substitution of Ti^4+^ at the Ti_5c_ sites by transition metals is energetically favored. Especially, the p dopings of Zn^2+^ and Y^3+^ for Ti^4+^ at interfaces are the most energetically favorable among the transition metals, which lead to improved interfacial stability. Electrostatic potential investigations revealed that the potential drop in the Zn- and Y-substituted interfaces is notably steeper than that in the other interfaces, indicating that the substituents are more efficient in separating the carriers. The relatively large ionic radius of Y^3+^ and small ionic radius of Zn^2+^ lead to distortions in the ELF calculations. The calculated absorption spectra indicate that the transition metal-substituted CH_3_NH_3_PbI_3_/TiO_2_ interface retains an enhanced visible light photocatalytic ability owing to the decreased transition energies. Closer comparisons between pristine and substituted CH_3_NH_3_PbI_3_/TiO_2_ indicate that the interfacial band gaps can be tuned by n, p, and isovalent substituents using the selected transition metals. Due to their optimal band alignment, the Nb^5+^ and W^6+^ substituted interface have better device performance. Theoretical studies predict that the varied mechanisms depending on transition metal substations will exert different effects on properties of CH_3_NH_3_PbI_3_/TiO_2_ interfaces. Our calculations explain why transition metals *M* (*M* = Zn^2+^, Y^3+^, Zr^4+^, Nb^5+^, Ru^4+^, W^6+^) could enhance device performance and why it is helpful for the potential commercialization of planar heterojunction PSC.

## Figures and Tables

**Table 1:** Calculated lattice parameters of TiO_2_ and MAPbI_3_ and deviation between experiment and simulation.

	Anatase TiO_2_		Tetragonal -MAPbI_3_	
	*a*	*c*	*a*	*c*
Experimental	3.785	9.514	8.80	12.685
Calculated	3.79	9.53	8.80	13.05
Deviation	0.13%	0.17%	-	2.8%

**Table 2:** Interfacial binding energy (in eV) and lattice mismatch of the CH_3_NH_3_PbI_3_/TiO_2_ interfaces.

	CH_3_NH_3_I/TiO_2_	PbI_2_/TiO_2_	CH_3_NH_3_I/TiO_2_ With Rotation	PbI_2_/TiO_2_ With Rotation
Binding energy	2.16	2.07	0.00	1.44
Lattice mismatch	−12.0%	−12.0%	−13.8%	−13.8%
Charge transfer	−0.29	−0.28	−0.16	−0.16

**Table 3:** Total energy (in eV) of CH_3_NH_3_PbI_3_/TiO_2_ with substitution of one Nb^5+^ for Ti^4+^.

Position	Surface (Ti_5c_)	Sub-Surface (Ti_6c_)	Inner-Surface (Third Ti Layer)	Inner-Surface (Fourth Ti Layer)
Total energy	−1401.71	−1401.94	−1401.52	−1401.72

**Table 4:** Interfacial binding energy (in eV) of the Nb-substituted and pristine CH_3_NH_3_PbI_3_/TiO_2_ interfaces.

	Ti_5c_ Site		Ti_6c_ Site		CH_3_NH_3_I/TiO_2_	PbI_2_/TiO_2_
	CH_3_NH_3_I/TiO_2_	PbI_2_/TiO_2_	CH_3_NH_3_I/TiO_2_	PbI_2_/TiO_2_		
Binding energy	3.59	3.05	2.77	2.54	0.09	0.00
Charge transfer	−0.44	−0.28	−0.26	−0.15	−0.29	−0.28
